# Incidence of somnolence and dizziness induced by mirogabalin and pregabalin under opioid treatment: a single-center observational study

**DOI:** 10.1186/s40780-025-00464-z

**Published:** 2025-07-01

**Authors:** Hitoshi Iwasaki, Hiroshi Kato, Takenao Koseki, Masashi Kondo, Shigeki Yamada

**Affiliations:** 1 Department of Pharmacotherapeutics and Informatics, Fujita Health University School of Medicine, 1–98 Dengakugakubo, Kutsukake-cho, Toyoake, 470–1192 Aichi Japan; 2 Department of Development and Education of Clinical Research, Fujita Health University School of Medicine, Toyoake, Aichi Japan; 3 Department of Regulatory Science for Evaluation & Development of Pharmaceuticals & Devices, Fujita Health University Graduate School of Medical Sciences, Toyoake, Aichi Japan; 4 Department of Respiratory Medicine, Fujita Health University School of Medicine, Toyoake, Aichi Japan

**Keywords:** Mirogabalin, Pregabalin, Somnolence, Dizziness, Opioid

## Abstract

**Background:**

The gabapentinoids pregabalin and mirogabalin are utilized to treat neuropathic pain, especially in patients with cancer receiving opioid analgesics. Pregabalin combined with strong opioids increases somnolence and dizziness, while mirogabalin causes fewer central adverse events. This study aimed to determine whether mirogabalin leads to a lower incidence of somnolence and dizziness than pregabalin in patients with cancer receiving strong opioids.

**Methods:**

We analyzed inpatients with cancer treated with mirogabalin or pregabalin along with strong opioids at Fujita Health University Hospital (April 2019–December 2023) and assessed cumulative incidence rates, hazard ratios (HRs) for somnolence and dizziness occurrence, and changes in morphine milligram equivalents (MMEs).

**Results:**

Among the 89 patients included in the analysis (mirogabalin: 39, pregabalin: 50), the median time to somnolence and dizziness was significantly shorter in the mirogabalin group than in the pregabalin group (8.0 vs. 17.0 days, *p* = 0.039). The multivariable Cox proportional regression model showed a higher risk with mirogabalin, although with no significance (HR: 1.74, *p* = 0.117). MMEs increased in the pregabalin group but not in the mirogabalin group.

**Conclusions:**

Mirogabalin and pregabalin contribute to somnolence and dizziness in patients receiving strong opioids, necessitating careful monitoring.

**Supplementary Information:**

The online version contains supplementary material available at 10.1186/s40780-025-00464-z.

## Background

Pregabalin and mirogabalin are standard therapies in neuropathic pain treatment. While effective, their use is associated with safety concerns, with the most common adverse events (AEs) being peripheral edema and increased weight, as well as central nervous system (CNS) depression, leading to somnolence and dizziness [1, 2]. Most of these AEs emerge within 3–4 weeks of treatment with pregabalin—within 1–2 weeks for somnolence and dizziness [3]—significantly reducing patient compliance and sometimes leading to discontinuation before the therapeutic effect can be achieved. The efficacy of pregabalin and mirogabalin in treating neuropathic pain is attributed to their modulation of glutamate release from hyperexcited neurons via binding to the α2δ subunit of voltage-gated calcium channels, which regulates calcium influx to presynaptic terminals [4]. Mirogabalin, first approved in Japan for peripheral neuropathic pain treatment in January 2019, shows potent and long-lasting analgesic effects by strongly binding and slowly dissociating from the α2δ-1 subunit. It also induces a lower level of CNS-specific AEs owing to its low affinity and rapid dissociation from the α2δ-2 subunit compared with pregabalin [5, 6].

Patients with moderate-to-severe pain may receive strong opioids during treatment; however, neuropathic cancer pain often resists opioid treatment, necessitating adjuvant drugs in combination, such as antidepressants and anticonvulsants, which have demonstrated efficacy [7]. Consequently, patients with cancer receiving gabapentinoids, including pregabalin and mirogabalin, for neuropathic pain are commonly prescribed opioids that exert CNS inhibitory effects, a combination presumed to enhance somnolence and dizziness [8]. Indeed, pregabalin is associated with increased somnolence and dizziness frequency, especially in co-administration with strong opioids [9, 10]. However, data on the influence of mirogabalin on CNS inhibitory effects in patients receiving opioids remain limited.

Recently, a pharmacovigilance study aimed at detecting drug-associated AE signals using the Japanese Adverse Drug Event Report database indicated that strong opioid analgesics increased the occurrence of somnolence when combined with pregabalin but not with mirogabalin [11]. Thus, mirogabalin may be more suitable than pregabalin, even when combined with strong opioids. However, this study utilized a spontaneous reporting database, and the findings require validation in clinical cohort studies comparing mirogabalin and pregabalin.

In this retrospective cohort study, we investigated the occurrence of somnolence and dizziness in patients receiving strong opioid analgesics and compared the differences in the frequency of these AEs between combinations with mirogabalin and pregabalin.

## Methods

### Study design and patient selection

Patients receiving mirogabalin or pregabalin while undergoing opioid analgesic treatment (oral or transdermal) during hospitalization at Fujita Health University Hospital between April 2019 and December 2023 were surveyed in this retrospective observational study. Patients who used opioid analgesics only for rescue purposes without baseline treatment were excluded.

### Data collection

The data were collected from electronic medical records of Fujita Health University Hospital. The baseline day of the first mirogabalin or pregabalin treatment was defined as day 1. The observation period was defined as the time until the occurrence of any of the following events, during which information on the onset of somnolence and dizziness was collected: discontinuation of mirogabalin or pregabalin (including switching to the other agent), discontinuation of oral/transdermal opioid analgesic treatment, and discharge from the hospital or death. Adverse events were extracted from the medical records as evaluated by physicians, nurses, pharmacists, and other medical staff within the scope of their normal duties. Patients who already had symptoms of somnolence or dizziness when starting pregabalin or mirogabalin were included if they reported “increased symptoms.” Age, sex, and body-mass index (BMI) data were collected, along with factors that may influence the occurrence of somnolence and dizziness, such as creatinine clearance, baseline opioid dosage (morphine milligram equivalents; MMEs), and mirogabalin and pregabalin dosages. Data on the use of non-steroidal anti-inflammatory drugs (NSAIDs) or acetaminophen at baseline, as well as the MMEs and mirogabalin and pregabalin dosages at the time of somnolence and dizziness, were also collected. MMEs were converted to opioid doses according to the conversion factor table by referring to the Centers for Disease Control and Prevention standard conversion factor, clinical guidelines for the pharmacological management of cancer pain in 2020 published by The Japanese Society of Palliative Medicine, and package product labeling of each opioid analgesic in Japan as previously reported (Additional file 1) [12].

### Statistical analysis

Patient characteristics are presented as medians and interquartile ranges for continuous variables and counts with percentages for categorical variables. Kaplan–Meier estimation calculated the cumulative incidence rate for each group. The difference in the cumulative incidence rate between the two groups was confirmed using the log-rank test. Cox proportional hazards regression analysis was performed to estimate the hazard ratio (HR) of the mirogabalin group relative to the pregabalin group for the time of somnolence and dizziness. The covariates selected to avoid overfitting were age group (≥ 65 years old/ < 65 years old), sex, presence of renal impairment (≤ creatinine clearance (CLcr) 60 mL/min/ > CLcr 60 mL/min), and baseline MMEs (per 10 mg unit). The between-group comparison of changes in MMEs from baseline to the occurrence of somnolence and dizziness was performed using the Mann–Whitney *U* test. Results were considered statistically significant at *p*-values < 0.05. We performed statistical analyses using EZR (Saitama Medical Center, Jichi Medical University, Saitama, Japan), a graphical user interface for R (The R Foundation for Statistical Computing, Vienna, Austria, version 4.2.2). More precisely, it is a modified version of R Commander (version 1.61) designed to add statistical functions frequently used in biostatistics [13].

## Results

### Patient characteristics

**Fig. 1:**
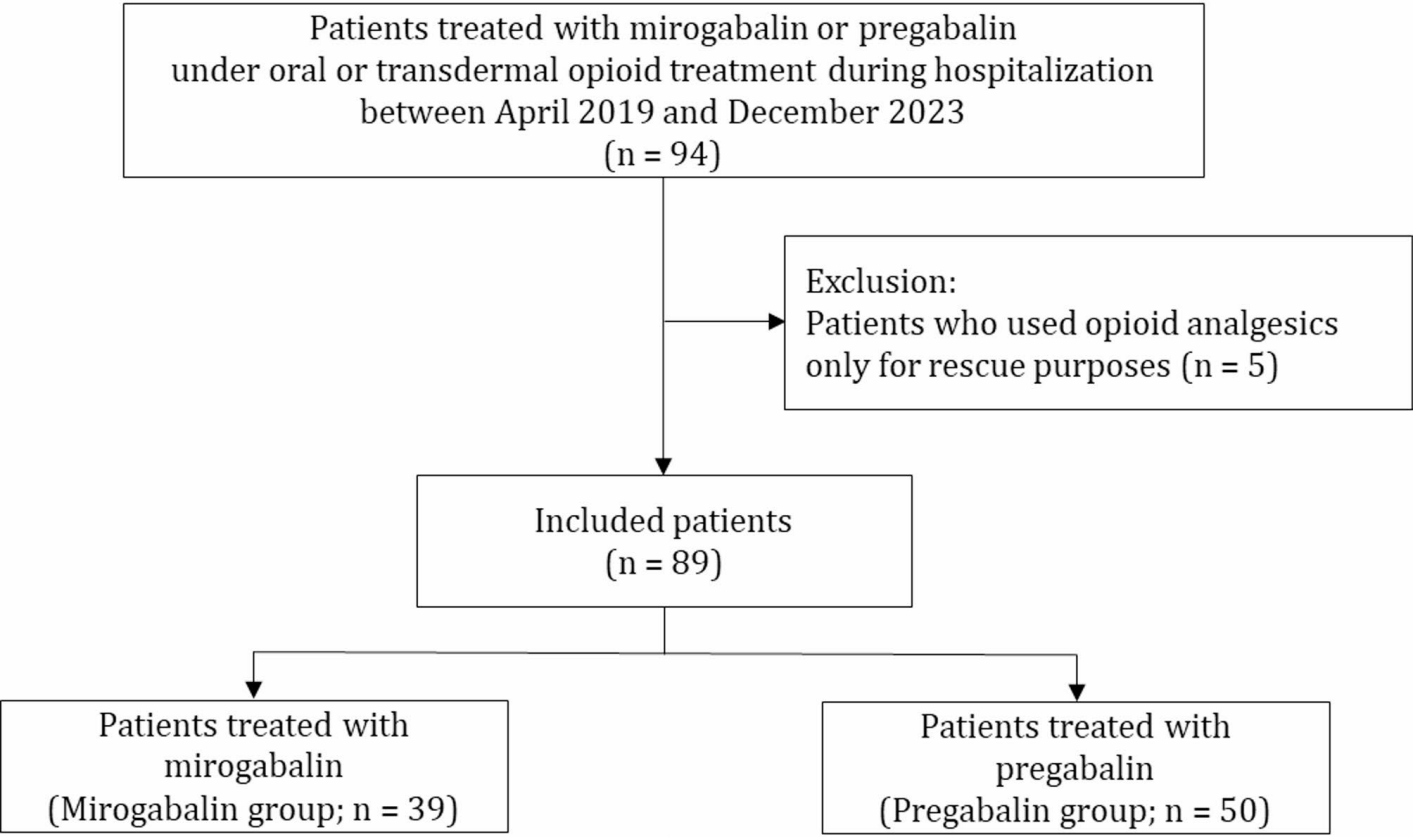
Patient selection flowchart

Of the 94 patients surveyed, five were excluded owing to opioid analgesic use for rescue purposes, leaving 89 included in this study. The included patients were divided into two groups: those who started receiving mirogabalin (mirogabalin group; 39 patients) and those who started pregabalin (pregabalin group; 50 patients) (Fig. 1).

The baseline characteristics of the patients are listed in Table 1. There were no substantial differences between the mirogabalin and pregabalin groups in terms of median age (71.0 vs. 64.5 years), BMI (19.6 vs. 20.5 kg/m^2^), CLcr (66.3 vs. 78.7 mL/min), or the percentage of females (43.6 vs. 34.0%), respectively. At baseline, the median MMEs in the pregabalin group were approximately twice as high as those in the mirogabalin group (60 mg vs. 30 mg, respectively); however, this difference was not statistically significant. The majority of opioids—78.0% in the mirogabalin group and 89.3% in the pregabalin group—were administered orally. The proportion of patients using NSAIDs/acetaminophen was comparable between the mirogabalin (69.2%) and pregabalin (72.0%) groups. The most common initial dosage at the start was 10 mg for mirogabalin (76.9%) and 150 mg for pregabalin (34.0%). Additionally, a considerable number of patients started pregabalin at lower doses: 50 mg (26.0%) and 75 mg (26.0%).

**Table 1:** Patient baseline characteristics

	All *n* = 89	Mirogabalin *n* = 39	Pregabalin *n* = 50
Age, yrs	68.0 (57.0, 78.0)	71.0 (59.5, 81.0)	64.5 (57.0, 74.8)
Sex			
Male	55 (61.8)	22 (56.4)	33 (66.0)
Female	34 (38.2)	17 (43.6)	17 (34.0)
BMI, kg/m^2^	20.0 (18.2, 22.6)	19.6 (18,3 22.2)	20.5 (20.0, 22.3)
CLcr, mL/min	75.5 (46.8, 94.2)	66.3 (42.8, 91.4)	78.7 (52.6, 97.5)
Baseline MMEs	45.0 (20.0, 90.0)	30.0 (20.0, 82.5)	60.0 (22.5, 90.0)
Baseline opioid types			
Oxycodone hydrochloride hydrate (po)	55 (61.8)	24 (61.5)	31 (62.0)
Fentanyl citrate (td)	15 (16.9)	4 (10.3)	11 (22.0)
Hydromorphone hydrochloride (po)	10 (11.2)	4 (10.3)	6 (12.0)
Morphine sulfate hydrate (po)	6 (6.7)	4 (10.3)	2 (4.0)
Morphine hydrochloride hydrate (po)	1 (1.1)	1 (2.6)	0 (0.0)
Tapentadol hydrochloride (po)	1 (1.1)	1 (2.6)	0 (0.0)
Methadone (po)	1 (1.1)	1 (2.6)	0 (0.0)
Patients using NSAIDs/acetaminophen at baseline	63 (70.8)	27 (69.2)	36 (72.0)
Baseline gabapentinoid dose	N.A.	2.5 mg: 1 (2.6)	25 mg: 4 (8.0)
		5 mg: 7 (17.9)	50 mg: 13 (26.0)
		10 mg: 30 (76.9)	75 mg: 13 (26.0)
		20 mg: 1 (2.6)	100 mg: 3 (6.0)
			150 mg: 17 (34.0)

Data indicates median (25th– 75th percentiles) or number (%)

BMI, body mass index; CLcr, creatinine clearance; MMEs, morphine milligram equivalents, N.A, not applicable; NSAIDs, non-steroidal anti-inflammatory drugs; po, per os; td, transdermal

### Differences between Mirogabalin and Pregabalin in the occurrence of Somnolence and Dizziness

**Fig. 2:**
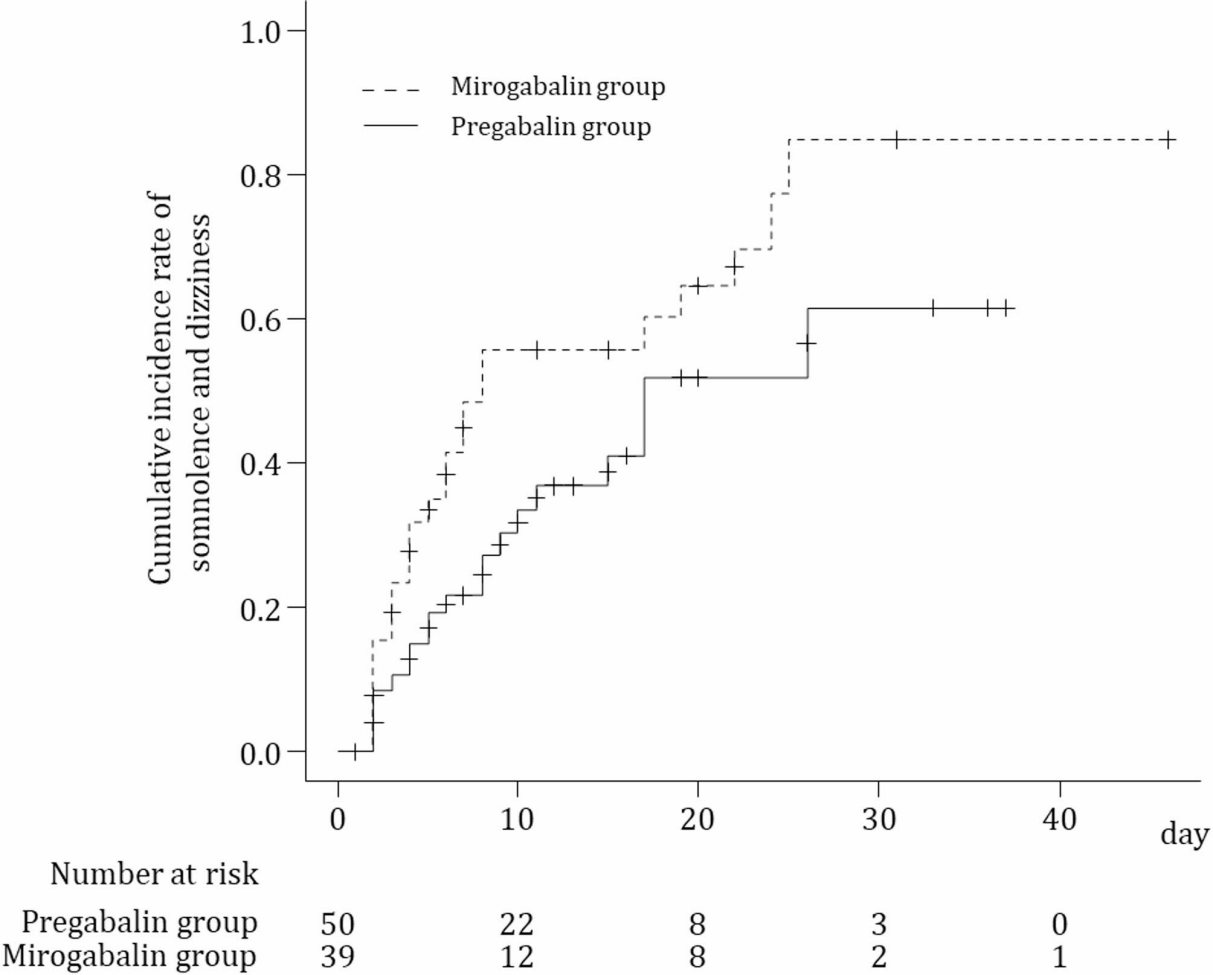
Kaplan–Meier curves of the occurrence of somnolence and dizziness. The vertical axis shows the cumulative incidence rate using the Kaplan–Meier estimation. The horizontal axis shows the number of days since the baseline day

The cumulative incidence curve for mirogabalin and pregabalin groups is shown in Fig. 2. The median [95% confidence interval (CI)] time of occurrence of somnolence and dizziness in the mirogabalin group was significantly shorter than that in the pregabalin group under the log-rank test (8.0 [5.0–22.0] vs. 17.0 [10.0–not reached], respectively; *p* = 0.039).

In the multivariable Cox proportional hazards regression analysis adjusted for age group (≥ 65 years and < 65 years), sex, presence of renal impairment (≤ CLcr 60 mL/min and > CLcr 60 mL/min), and baseline MMEs (per 10 mg unit), the mirogabalin group showed a higher risk of somnolence and dizziness than the pregabalin group (HR: 1.74 [95% CI 0.93–3.26], *p* = 0.117), although without significance. Furthermore, when CLcr was ≤ 60 mL/min, the risk of somnolence/dizziness tended to be higher in both groups (HR: 2.01 [95% CI 0.96–4.20], *p* = 0.065) (Table 2).

**Table 2:** Hazard ratio for occurrence of somnolence and Dizziness in patients treated with pregabalin/mirogabalin under opioid treatment

	HR	95%CI	*P*-value
Mirogabalin treatment (vs. pregabalin treatment)	1.74	0.93–3.26	0.117
≥ 65 years old (vs. < 65 years old)	0.90	0.43–1.88	0.771
Female (vs. male)	0.89	0.47–1.71	0.736
≤ CLcr 60 mL/min (vs. > CLcr 60 mL/min)	2.01	0.96–4.20	0.065
Baseline MMEs (per 10 mg unit)	1.00	0.98–1.02	0.793

CI, confidence interval; CLcr, creatinine clearance; HR, hazard ratio; MMEs, morphine milligram equivalents

### Changes in opioid dosage from baseline to occurrence of Somnolence and Dizziness

**Fig. 3:**
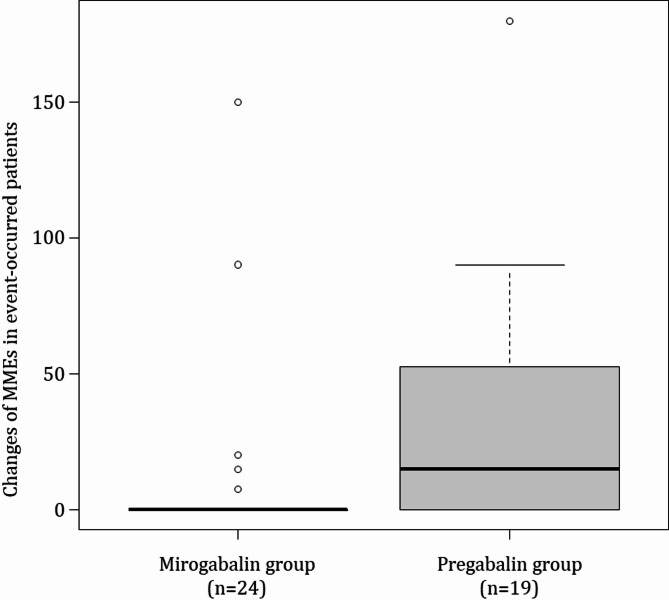
Changes in morphine milligram equivalents in patients with somnolence and dizziness in each group. The central line in each box represents the median, while the upper and lower edges indicate the first and third quartiles. The whiskers extend to 1.5 times the interquartile range, and any points beyond this range are considered outliers (denoted as dots). MMEs, morphine milligram equivalents

The occurrence of somnolence and dizziness might be influenced by an increase in opioid dosage. Therefore, we investigated the changes in MMEs from baseline to the occurrence of somnolence and dizziness. MMEs did not change in the mirogabalin group but increased in the pregabalin group up to the occurrence of somnolence and dizziness (median [interquartile range] = 15.0 [0–52.5], *p* = 0.014) (Fig. 3).

### Changes in Mirogabalin and Pregabalin doses from baseline to the occurrence of Somnolence and Dizziness

Dose changes of mirogabalin and pregabalin after the initiation of opioid co-administration might also influence the occurrence of somnolence and dizziness. Thus, we investigated the changes in mirogabalin and pregabalin dosage from baseline to the occurrence of somnolence and dizziness. In the mirogabalin group, 87.5% of the patients had no change in dosage from baseline to the occurrence of somnolence and dizziness. In the pregabalin group, while 68.4% of the patients had no change in dosage from baseline to the occurrence of somnolence and dizziness, 31.6% received increased doses (Table 3).

**Table 3:** Number of patients with changes in mirogabalin and pregabalin dosage from baseline to the time of somnolence and dizziness occurrences

	Mirogabalin group (*n* = 24)	Pregabalin group (*n* = 19)
No change	21 (87.5)	13 (68.4)
Increase	2 (8.3)	6 (31.6)
Decrease	1 (4.2)	0 (0.0)

### Subgroup analysis for differences in the occurrence of Somnolence and Dizziness between Mirogabalin and Pregabalin

To eliminate the potential influence of somnolence and dizziness occurring immediately after opioid initiation, the exacerbation of pre-existing somnolence and dizziness, and the effects of transdermal formulations (e.g., fentanyl), we conducted subgroup analyses limited to (1) patients who had been using opioids for at least one week prior to baseline (2), patients who did not exhibit somnolence or dizziness at baseline, and (3) patients who were using oral opioid formulations at baseline (Additional files 2–7). While the results should be interpreted with caution given that these are subgroup analyses, trends similar to those in the overall population were observed across all subgroups.

## Discussion

The neuropathic component in cancer pain responds more effectively to pregabalin than strong opioids; therefore, mirogabalin may also be effective [14]. Increased somnolence and dizziness have been reported following treatment with pregabalin in combination with strong opioids, but reports on mirogabalin remain limited [15]. Studies investigating and comparing the frequency of somnolence and dizziness in patients using strong opioids concomitantly with pregabalin or mirogabalin are also limited. In this study, we investigated the differences in the frequency of somnolence and dizziness in patients under strong opioids combined with mirogabalin or pregabalin.

Mirogabalin and pregabalin modulate glutamate release from hyperexcited neurons via binding to the α2δ subunit. Pregabalin binds non-selectively to α2δ-1 and α2δ-2 subunits, leading to CNS-AEs, whereas mirogabalin has a highly specific binding affinity for the α2δ subunit, with a slower dissociation rate for α2δ-1 than α2δ-2. This suggests that mirogabalin may exert fewer AEs than pregabalin [16]. According to a retrospective study, the incidence rates of somnolence and dizziness with mirogabalin are reduced in patients with lumbar disease presenting with neuropathic pain who switched from pregabalin to mirogabalin [17]. Despite the lower frequency of mirogabalin-related CNS-AEs due to pharmacological characteristics, in the present study, somnolence and dizziness tended to occur over a shorter period in the mirogabalin group than in the pregabalin group in combination with strong opioids, as indicated by the log-rank test. In the Cox proportional regression model, the HR of somnolence and dizziness in the mirogabalin group compared with the pregabalin group was 1.74 (95% CI 0.93–3.26, *p* = 0.117), which differed from the initial expectation that these AEs were less likely to occur in the mirogabalin group.

A previous retrospective cohort study comparing the occurrence of side effects in patients starting mirogabalin or pregabalin for neuropathic pain reported a higher HR for somnolence and dizziness with mirogabalin [18]. In addition, a randomized phase 2 trial including Asians directly compared the risk of adverse events with mirogabalin and pregabalin and reported that the risk ratios for somnolence and dizziness were similar between the two groups [19]. These findings, along with ours, indicate that increased incidence of somnolence and dizziness should be expected in patients receiving strong opioids, not only with pregabalin but also with mirogabalin. There was no significant difference in opioid dosage at the start of the pregabalin or mirogabalin combination in either group. Comparing the difference in opioid doses during the time to the event, the pregabalin group had a larger increase in opioid dose. Therefore, the difference in the increase of somnolence and dizziness observed between the mirogabalin and the pregabalin groups is not attributable to the increase in opioid dosage. As increases in baseline opioid doses are based on the efficacy and frequency of rescue doses and are not applied rapidly, it is unlikely that the incidence of somnolence or dizziness observed in this study was strongly influenced by opioid dose escalation in combination with pregabalin or mirogabalin. Furthermore, during the time to the event, a greater proportion of patients in the pregabalin group underwent gabapentinoid dose escalation than that in the mirogabalin group. Thus, the influence of gabapentinoid dose increases on the between-group differences in the incidence of somnolence and dizziness observed in this study is also considered to be limited.

In the adjusted Cox proportional regression model, the presence of impaired renal function was identified as a factor that influences the occurrence of somnolence and dizziness. More than 90% of mirogabalin and pregabalin are renally excreted as unchanged drugs, and their clearance is reduced in patients with renal impairment [20, 21]. Although this is only an adjusted factor and not an attempt to identify risk factors, caution is necessary concerning the occurrence of somnolence and dizziness in patients with impaired renal function receiving strong opioids and showing CLcr < 60 mL/min, regardless of combination with pregabalin or mirogabalin.

This study has some limitations. First, the potential influence of psychotropic and other drugs that tend to cause somnolence has not been ruled out. Second, this is a retrospective study based on data extracted from medical records; therefore, the results might be affected by the standards used by medical staff to record adverse events. Further multicenter observational studies that include more facilities and cases and incorporate prospective and well-designed data collection are required to enhance the generalizability of our findings.

## Conclusions

In conclusion, although we initially expected that mirogabalin would be associated with a lower incidence of somnolence and dizziness compared with pregabalin when used in combination with opioids, our findings demonstrated that mirogabalin did not reduce the occurrences of these events to a greater extent than pregabalin. Therefore, caution is necessary with both pregabalin and mirogabalin use regarding the increased occurrence and exacerbation of somnolence and dizziness in opioid-treated patients.

## Electronic supplementary material

Below is the link to the electronic supplementary material.

Supplementary Material 1

Supplementary Material 2

Supplementary Material 3

Supplementary Material 4

Supplementary Material 5

Supplementary Material 6

Supplementary Material 7

## Data Availability

No datasets were generated or analysed during the current study.

## Abbreviations

AE: Adverse event
BMI: Body-mass index
CI: Confidence interval
CLcr: Creatinine clearance
CNS: Central nervous system
HR: Hazard ratio
MME: Morphine milligram equivalent
N.A.: Not Applicable
NSAID: Non-steroidal anti-inflammatory drug
po: Per os
td: Transdermal
