# Supplementary material for: Incidence of somnolence and dizziness induced by mirogabalin and pregabalin under opioid treatment: a single-center observational study
Source: J Pharm Health Care Sci. 2025 Jul 1;11:54. doi: 10.1186/s40780-025-00464-z (PMC12220117; doi:10.1186/s40780-025-00464-z)
Supplement: Supplementary file 1 — Supplementary Material 1 [file 40780_2025_464_MOESM1_ESM.docx]

## Additional File 1

**Additional Table. Morphine milligram equivalents conversion list**

|  | MMEs |
| --- | --- |
| Morphine Sulfate Hydrate (po) | 1.0 |
| Morphine Hydrochloride Hydrate (po) | 1.0 |
| Oxycodone Hydrochloride Hydrate (po) | 1.5 |
| Hydromorphone Hydrochloride (po) | 5.0 |
| Tapentadol Hydrochloride (po) | 0.3 |
| Fentanyl Citrate (td) | 30.0 |
| Methadone (po) | 12.0 |

MMEs, morphine milligram equivalents; po, per os: td, transdermal.
