# Supplementary material for: Incidence of somnolence and dizziness induced by mirogabalin and pregabalin under opioid treatment: a single-center observational study
Source: J Pharm Health Care Sci. 2025 Jul 1;11:54. doi: 10.1186/s40780-025-00464-z (PMC12220117; doi:10.1186/s40780-025-00464-z)
Supplement: Supplementary file 2 — Supplementary Material 2 [file 40780_2025_464_MOESM2_ESM.docx]

## Additional File 2

**Additional Table. Hazard ratios for the occurrence of somnolence and dizziness in patients treated with pregabalin/mirogabalin under opioid treatment (subgroup of patients who had been using opioids for at least one week prior to baseline).**

|  | HR | 95% CI | *P*-value |
| --- | --- | --- | --- |
| Mirogabalin treatment  (vs. pregabalin treatment) | 1.86 | 0.87–3.98 | 0.109 |
| ≥ 65 years old  (vs. < 65 years old) | 0.79 | 0.33–1.91 | 0.605 |
| Female  (vs. male) | 1.07 | 0.50–2.27 | 0.863 |
| ≤ CLcr 60 mL/min  (vs. > CLcr 60 mL/min) | 2.20 | 0.90–5.43 | 0.086 |
| Baseline MMEs (per 10 mg unit) | 1.00 | 0.98–1.02 | 0.952 |

CI, confidence interval; CLcr, creatinine clearance; HR, hazard ratio; MMEs, morphine milligram equivalents.
