# Supplementary material for: Incidence of somnolence and dizziness induced by mirogabalin and pregabalin under opioid treatment: a single-center observational study
Source: J Pharm Health Care Sci. 2025 Jul 1;11:54. doi: 10.1186/s40780-025-00464-z (PMC12220117; doi:10.1186/s40780-025-00464-z)
Supplement: Supplementary file 3 — Supplementary Material 3 [file 40780_2025_464_MOESM3_ESM.docx]

## Additional File 3

**Additional Table. Hazard ratios for the occurrence of somnolence and dizziness in patients treated with pregabalin/mirogabalin under opioid treatment (subgroup of patients who did not exhibit somnolence or dizziness at baseline).**

|  | HR | 95% CI | *P*-value |
| --- | --- | --- | --- |
| Mirogabalin treatment  (vs. pregabalin treatment) | 1.68 | 0.86–3.27 | 0.128 |
| ≥ 65 years old  (vs. < 65 years old) | 0.86 | 0.39–1.87 | 0.699 |
| Female  (vs. male) | 1.00 | 0.50–2.00 | 0.995 |
| ≤ CLcr 60 mL/min  (vs. > CLcr 60 mL/min) | 1.83 | 0.81–4.15 | 0.145 |
| Baseline MMEs (per 10 mg unit) | 1.00 | 0.98–1.02 | 0.982 |

CI, confidence interval; CLcr, creatinine clearance; HR, hazard ratio; MMEs, morphine milligram equivalents.
