# Supplementary material for: Incidence of somnolence and dizziness induced by mirogabalin and pregabalin under opioid treatment: a single-center observational study
Source: J Pharm Health Care Sci. 2025 Jul 1;11:54. doi: 10.1186/s40780-025-00464-z (PMC12220117; doi:10.1186/s40780-025-00464-z)
Supplement: Supplementary file 4 — Supplementary Material 4 [file 40780_2025_464_MOESM4_ESM.docx]

## Additional File 4

**Additional Table. Hazard ratios for the occurrence of somnolence and dizziness in patients treated with pregabalin/mirogabalin under opioid treatment (subgroup of patients who were using oral opioid formulations at baseline).**

|  | HR | 95% CI | *P*-value |
| --- | --- | --- | --- |
| Mirogabalin treatment  (vs. pregabalin treatment) | 1.60 | 0.81–3.17 | 0.174 |
| ≥ 65 years old  (vs. < 65 years old) | 1.07 | 0.49–2.33 | 0.865 |
| Female  (vs. male) | 1.07 | 0.52–2.19 | 0.855 |
| ≤ CLcr 60 mL/min  (vs. > CLcr 60 mL/min) | 1.62 | 0.73–3.57 | 0.233 |
| Baseline MMEs (per 10 mg unit) | 1.00 | 0.98–1.02 | 0.838 |

CI, confidence interval; CLcr, creatinine clearance; HR, hazard ratio; MMEs, morphine milligram equivalents.
