# Supplementary material for: Incidence of somnolence and dizziness induced by mirogabalin and pregabalin under opioid treatment: a single-center observational study
Source: J Pharm Health Care Sci. 2025 Jul 1;11:54. doi: 10.1186/s40780-025-00464-z (PMC12220117; doi:10.1186/s40780-025-00464-z)

## Additional File 6

**Additional Figure. Kaplan–Meier curves of the occurrence of somnolence and dizziness for the subgroup of patients who did not exhibit somnolence or dizziness at baseline. The vertical axis shows the cumulative incidence rate using the Kaplan–Meier estimation. The horizontal axis shows the number of days since the baseline day.**


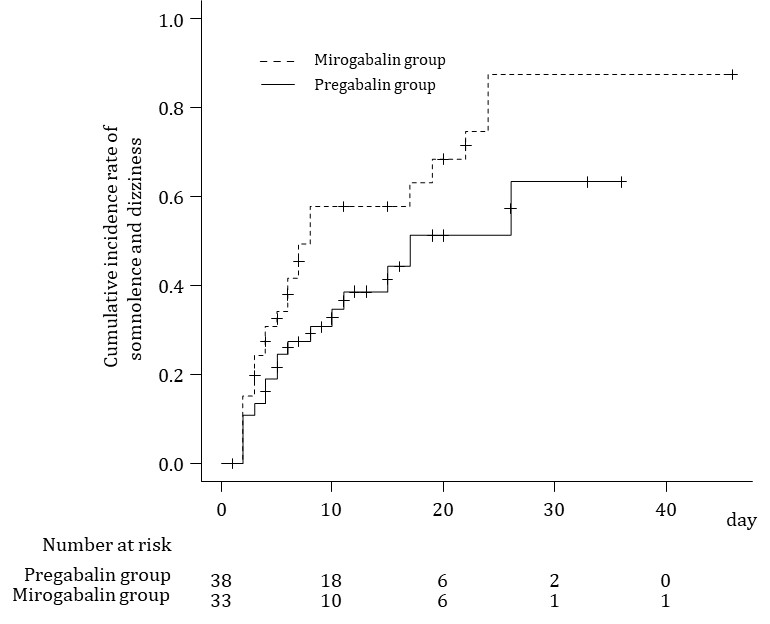

Supplement: Supplementary file 6 — Supplementary Material 6 [file 40780_2025_464_MOESM6_ESM.docx]
